# Minimally invasive pedicle screw placement with image-guided navigation in cervical spine injuries

**DOI:** 10.1007/s00701-025-06680-8

**Published:** 2025-09-25

**Authors:** Clemens Weber, Kjell Akre, Cecilia Avellan, Maziar Behbahani, David Werner

**Affiliations:** Department of Neurosurgery, Stavanger University Hospital, P.O. Box 8100, 4068 Stavanger, Norway

**Keywords:** Cervical fracture, MIS, Navigation

## Abstract

**Introduction:**

MIS pedicle screw placement is a novel technique for the management of unstable injuries of the cervical spine. This study aims to evaluate the feasibility of MIS pedicle screw placement and to compare perioperative, postoperative and radiological outcomes between MIS and conventional open approaches.

**Methods:**

This single-centre cohort study included patients with unstable injuries of the cervical spine treated with open approaches for pedicle or lateral mass screw fixation or MIS approaches for pedicle screw fixation. Perioperative and postoperative variables were compared. All screw positions were classified according to the Bredow classification.

**Results:**

Twenty patients with unstable injuries of the cervical spine were included, 10 undergoing conventional open posterior fixation (87 screws) and 10 undergoing MIS cervical pedicle screw fixation (48 screws). In the MIS group, significantly fewer vertebrae were instrumented (2.4 vs. 4.5; *p* = 0.008) and significantly fewer screws placed (4.8 vs. 8.7; p = 0.009). Operative time was significantly shorter in patients operated with MIS approach (183 vs. 132 min; p = 0.020). Also, there was a significant reduction in blood loss per surgery in patients operated with a MIS approach compared to an open approach (145 vs. 891 ml; p = 0.002). Out of 87 pedicle and lateral mass screws placed with an open approach 99% were classified as Bredow grade 1 or 2. All 48 screws placed with a MIS approach were rated as Bredow grade 1 or 2.

**Conclusions:**

This feasibility study provides preliminary evidence that surgery with MIS approach with navigated pedicle screws may be associated with reduced length of surgery and intraoperative blood loss compared to open surgery. Radiological evaluation of screw placement showed a good positioning with both open and minimally invasive approach. The results highlight the need for further investigation in larger, controlled trials to more rigorously evaluate the potential benefits and risks of this approach.

## Introduction

Unstable cervical spine injuries may be managed with anterior plating, posterior screw-rod constructs or a combination of both [16]. Posterior instrumentation is typically performed via open approaches, utilizing lateral mass or pedicle screws [18]. However, open posterior approaches are associated with significant approach-related morbidity including infections, wound dehiscence and paraspinal muscle atrophy [20, 21].

Numerous studies have demonstrated the biomechanical superiority of pedicle screws compared to lateral mass screws in the cervical spine, providing greater construct stability, reduced risk of implant failure, and allowing shorter constructs with potentially reduced morbidity and implant costs [7–9, 19]. However, the placement of cervical pedicle screws entails a higher risk of injury to the spinal cord, the cervical nerve roots and the vertebral arteries.

Minimally invasive surgery (MIS), commonly used in thoracolumbar spine injuries, offers advantages such as reduced blood loss, shorter hospital stays and earlier pain relief [5]. MIS utilizes small incisions with minimal soft tissue dissection, leading to reduced blood loss, and shorter operative times. This reduced invasiveness translates into shorter hospital stay, reduced pain levels and a faster recovery for the patients [10].

In recent years image-guided neuronavigation with intraoperative 3D-imaging such as computed tomography (CT) has been implemented in spine surgery to increase accuracy of screw placement and reduce re-operation rates. Automated neuronavigation workflows with intraoperative 3D-imaging performed with intraoperative CT enables a safer screw placement also in smaller bony structures such as the pedicles of the cervical spine [2]. In addition, image-guided neuronavigation carries the potential to implant cervical pedicle screws with a MIS approach possibly leading to reduced soft tissue damage, reduced blood loss and less postoperative pain [12, 14, 15]. It is unknown whether minimally invasive approaches in the cervical spine are also leading to similar benefits as in the thoracolumbar spine.

The current study aims to evaluate the feasibility of MIS pedicle screw placement in subaxial cervical spine injuries and to compare perioperative, postoperative and radiological outcomes between MIS and conventional open approaches.

## Methods

### Study design

This feasibility study included patients with unstable injuries of the cervical spine treated with open approaches for pedicle or lateral mass screw fixation or MIS approaches for pedicle screw fixation. Data on patients treated with an open approach were collected retrospectively from electronic medical records. Data for the MIS group were collected prospectively at the time of surgery and during follow-up. The study was approved by the institutional data protection officer as a quality assurance project (eProtokoll ID: 4993). Consent to participate has been waived according to the Norwegian Healthcare Personnel Act.

### Study population

Adult patients (age ≥ 18 years) with unstable injuries of the subaxial cervical spine (C3-C7) requiring surgical stabilization were included. Patients with injuries requiring posterior decompression (i.e. cervical laminectomy for decompression of the spinal cord) were not included. Injuries were confirmed by preoperative computed tomography (CT) and, when indicated, magnetic resonance imaging (MRI) before surgery. MIS cervical pedicle screw placement was introduced in January 2024. All surgeons involved in these surgeries were board-certified neurosurgeons with focus on spinal surgery and trained in MIS pedicle screw placement of the thoracolumbar spine.

### Surgical techniques

All surgeries were performed under general anesthesia with antibiotic prophylaxis. Patients were positioned in prone position on an operating table with a radiolucent carbon fiber tabletop. The patients’ head was fixed in a radiolucent Mayfield head clamp.

#### Open approach

A midline incision was made, the subcutaneous tissue was divided, and the fascia opened. Paraspinal muscles were detached to expose the laminae and the lateral masses. Neuronavigation or fluoroscopy guidance were utilized in all surgeries. When neuronavigation was used, a spinous process clamp was placed and a reference frame for the neuronavigation application (Navigation Software Spine and Trauma, Brainlab, Munich, Germany) was attached. A 3D-image was obtained with a robotic cone-beam CT imaging system (Loop-X, Brainlab, Munich, Germany) and transferred to the mobile neuronavigation unit (Curve, Brainlab, Munich Germany). Image fusion with preoperative CT or MRI was optional. If fluoroscopy guidance was utilized a mobile c-arm was used to identify the screw trajectories. Pedicle or lateral mass screws were implanted according to the trajectories identified with neuronavigation or fluoroscopy guidance. Rods were mounted to the screws and locked with locking screws. The wound was closed in a standard fashion. Postoperative CT was performed to confirm screw placement.

#### MIS approach

**Fig. 1:**
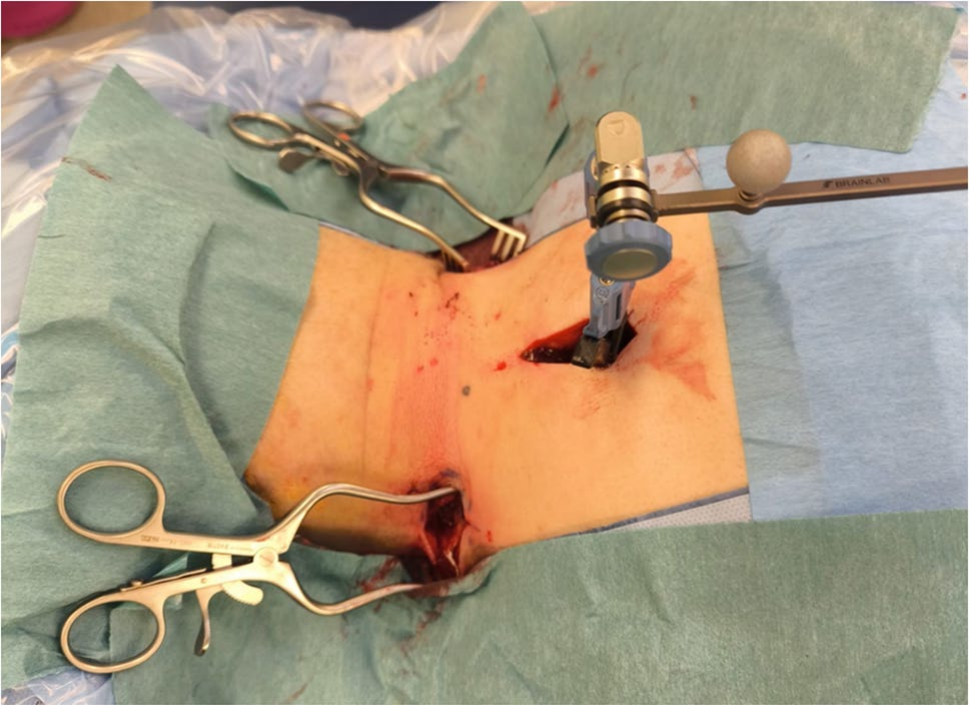
Intraoperative image with skin incisions for cervical pedicle screws and patient reference in the midline

A short midline incision was made cranially or caudally to the injury site. A spinous process clamp was placed and the reference frame for the neuronavigation application (Navigation Software Spine and Trauma, Brainlab, Munich, Germany) was attached. A 3D-image was obtained with the robotic imaging system (Loop-X, Brainlab, Munich, Germany) and transferred to the mobile neuronavigation unit (Curve, Brainlab, Munich, Germany). Image fusion with preoperative CT or MRI was optional. Screw trajectories for the pedicle screws and skin incisions were planned based on the navigation imaging. The trajectories for cervical pedicle screws were angled up to 45 degrees and skin incisions could be on the lateral aspect of the neck (Fig. 1). Navigated drill guide was used to drill the screw canals through the pedicles and k-wires were inserted. Cannulated screws were then implanted guided by neuronavigation. A robotic CBCT scan was performed intraoperatively to confirm screw positions. Rods were then mounted to the screws and locked with locking screws. The incisions were closed in a standard fashion.

### Radiological assessment

Postoperative CT or intraoperative CBCT scans were used to evaluate screw positioning. All screw positions were classified according to the Bredow classification which is a reliable classification of cervical spine instrumentation with various screw types [4]. Screw positions were graded into grade 1 (pedicle or lateral mass wall perforation < 1 mm), grade 2 (perforation < 2 mm), grade 3 (perforation < 3 mm), grade 4 (perforation < 4 mm) and grade 5 (perforation > 4 mm and/or obstruction of transverse foramen by more than half a screw diameter) [4].

### Statistics

Standard descriptive statistics summarized patient and surgical data. Continuous variables were reported as means with standard deviations, categorical variables as frequencies and proportions. Comparisons of continuous variables were performed using independent t-tests, qualitative variables were compared using chi-square tests. A p-value < 0.05 was considered statistically significant. Due to the small sample size and non-randomized design, this study was not powered for multivariable analysis or adjustment for confounding variables. The statistical findings should therefore be interpreted as exploratory.

## Results

A total of 20 patients with unstable injuries of the cervical spine were included, with 10 patients undergoing conventional open posterior fixation (87 screws) and 10 patients undergoing MIS cervical pedicle screw fixation (48 screws). There were no statistically significant differences in the patient characteristics between both groups (Table 1).

**Table 1:** Characteristics of patients with unstable injuries of the cervical spine operated with open or minimally invasive approach

	All patients (*n* = 20)	Open approach (*n* = 10)	MIS approach (*n* = 10)	*p*-value
Age in years, median (IQR)	65.5 (43.0–73.5)	69.5 (55.0–77.5)	60.0 (38.0–69.5)	0.656
Male sex, *n* (%)	13 (65)	5 (50)	8 (80)	0.349
BMI, mean (SD)	26.4 (5.0)	27.4 (6.9)	25.4 (2.0)	0.385
Spinal cord injury, n (%)	6 (30)	4 (40)	2 (20)	0.628
ASIA grade, *n* (%)				
A	1 (5)	0	1 (10)	0.471
B	0	0	0	
C	3 (15)	2 (20)	1 (10)	
D	2 (10)	2 (20)	0	
E	14 (70)	6 (60)	8 (80)	

*MIS* minimally invasive surgery, *BMI* body mass index, *ASIA* American Spinal Injury Association, *IQR* interquartile range, *SD* standard deviation

The surgical variables are presented in Table 2. In the MIS group, significantly fewer vertebrae were instrumented (2.4 vs. 4.5; p = 0.008) and significantly fewer screws placed (4.7 vs. 8.7; p = 0.009). Operative time was significantly shorter in patients operated with MIS approach (183 vs. 132 min; p = 0.020). Also, there was a significant reduction in blood loss per surgery in patients operated with a MIS approach compared to an open approach (145 vs. 891 ml; p = 0.002). There were no differences in hospital length of stay between the groups. In the open approach group, there were 2 readmissions within 30 days with superficial wound infections needing revision. None of the patients with a MIS approach were readmitted within 30 days.

**Table 2:** Surgical variables of patients with unstable injuries of the cervical spine operated with open or minimally invasive (MIS) approach

	All patients (*n* = 20)	Open approach (*n* = 10)	MIS approach (*n* = 10)	*p*-value
Use of navigation, n (%)	18 (90)	8 (80)	10 (100)	0.474
Instrumented vertebrae, mean (SD)	3.5 (1.9)	4.5 (2.1)	2.4 (0.8)	*0.008*
Number of screws, mean (SD)	6.8 (3.5)	8.7 (3.8)	4.7 (1.7)	*0.009*
Number of pedicle screws, mean (SD)	4.5 (2.3)	4.1 (2.8)	4.7 (1.7)	0.512
Number of lateral mass screws, mean (SD)	2.2 (2.9)	4.4 (2.7)	0	-
Length of surgery in minutes, mean (SD)	157 (51)	183 (55)	132 (32)	*0.020*
Blood loss in ml, mean (SD)	518 (586)	891 (629)	145 (140)	*0.002*
Length of stay in days, mean (SD)	6.4	6.7	6.2	0.886
Readmission within 30 days, *n* (%)	2 (10)	2 (20)	0	-
Reoperation within 30 days, n (%)	2 (10)	2 (20)	0	-

*MIS* minimally invasive surgery, *SD* standard deviation

**Fig. 2:**
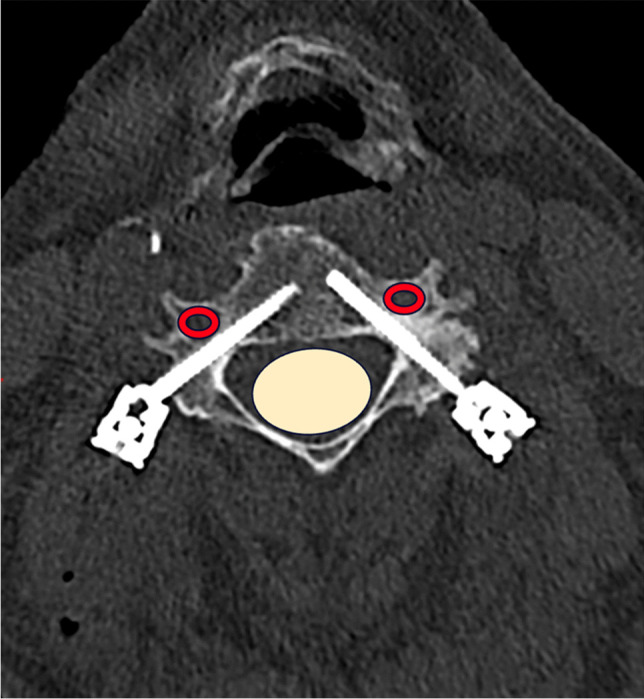
Postoperative CT image in the axial plane showing cervical pedicle screws in place, the vertebral arteries are marked in red, the spinal cord in yellow

All screw positions (*n* = 135) were evaluated according to the Bredow classification (Table 3). Out of 87 pedicle and lateral mass screws placed with an open approach 99% were classified as Bredow grade 1 or 2. All 48 screws placed with a MIS approach were rated as Bredow grade 1 or 2. Figure 2 shows a postoperative CT scan of pedicle screws rated as Bredow grade 1.

**Table 3:** Radiological grading of cervical screw positions according to the Bredow classification

	All patients (*n *= 20)	Open approach (*n* = 10)	MIS approach (*n* = 10)	*p*-value
Number of screws, *n* (%)	135 (100)	87 (100)	48 (100)	
Bredow grade 1	111 (82)	68 (78)	43 (90)	0.097
Bredow grade 2	23 (17)	18 (21)	5 (10)	0.129
Bredow grade 3	1 (1)	1 (1)	0	-
Bredow grade 4	0	0	0	-
Bredow grade 5	0	0	0	-

*MIS* minimally invasive surgery

## Discussion

This preliminary analysis of patients operated for unstable injuries of the subaxial cervical spine shows that surgery with a MIS approach with navigated pedicle screws can lead to shorter constructs with fewer instrumented vertebrae and screws. This translates into a significant reduction in length of surgery and intraoperative blood loss compared to open surgery with lateral mass and pedicle screws. Radiological evaluation of screw placement according to the Bredow classification for cervical spine screws showed a good positioning with both open and minimally invasive approach.

Navigated, posterior pedicle screw placement with a MIS approach is a relatively new technique for the treatment of unstable cervical spine injuries. The current analysis of an initial group of 10 patients treated with this approach showed that surgeons tend to shorter constructs with less segments involved and a reduced number of screws than with an open approach. Based on this findings the utilization of more biomechanically robust pedicle screws in these patients may enable smaller surgical solutions with short segment constructs [12]. The muscle-splitting MIS approach preserves paraspinal muscle attachments, potentially reducing muscle atrophy and improving postoperative posture. Another advantage could be a reduction of wound infections, in the current study there were no wound infections reported within 30 days after surgery. Open posterior fixation of the cervical spine is still associated with a surgical site infection of up to 25% [1]. Another finding of this analysis was the significant reduction in length of surgery which may be associated with the MIS approach with reduced time for approach and closing of the wounds. Also, the intraoperative blood loss was significantly reduced from a mean of 891 ml with an open approach to a mean of 145 ml with an MIS approach. Reduced blood loss can lead to shorter hospital stays, reduced treatment costs and reduced mortality in spine surgery [11].

Percutaneous pedicle screw placement based on fluoroscopy can be demanding in the cervical spine with up to 25% of inaccurately placed screws with the potential risk of severe complications such as spinal cord, nerve root and vertebral artery injury [13]. The development of neuronavigation systems with intraoperative imaging potentially improves the accuracy of pedicle screw placement also in the cervical spine. One needs to be aware of the inherent risks when utilizing neuronavigation in this highly mobile part of the spinal column, especially the potential for vertebral body shift and rotation when instrumenting and exerting pressure on the bony structures [17]. In the current study, all percutaneously placed screws demonstrated excellent or good positioning on postoperative imaging, as classified according to the Bredow system for cervical spine screws. [4]. Two recent studies from Germany reported similar accuracies above 80% of excellent or good screw positioning when using intraoperative image guided neuronavigation for MIS implantation of cervical pedicle screws [3, 14]. Our analysis is based on fewer surgeries and screws but showed an even higher accuracy.

## Limitations

This study has certain limitations such as the low number of patients which does not allow us to draw any definite conclusions. However, the technique described and evaluated in this study is quite new, and the current analysis is the first one comparing MIS to open approach in cervical screw placement. New surgical interventions should be evaluated in appropriately powered, randomized trial. However, initiating a large-scale, high-quality randomized trial is challenging due to uncertainties around the stability and standardization of the interventions, selection and measurement of relevant clinical and patient‐reported outcomes, and issues surrounding patient recruitment such as clinician and patient equipoise, therefore comparative studies evaluating initial experiences with a new surgical intervention may help to inform successful main trial design and conduct [6].

An important limitation is the inconsistent use of imaging guidance between groups. While all MIS procedures used intraoperative CT-based navigation, some open surgeries relied on fluoroscopy alone. This discrepancy may have influenced the accuracy outcomes and should be accounted for in future comparative studies.

Another limitation is the necessity of an advanced neuronavigation system for the accurate placement of percutaneous implants in the cervical spine. Furthermore, a learning curve is associated with the adoption of MIS techniques, which may affect outcomes during the initial phase of implementation. In addition, these systems are still expensive and not widely available.

## Conclusion

The results of this analysis indicate that a navigated, minimal-invasive approach with cervical pedicle screw placement is feasible and can lead to shorter constructs with less instrumented vertebrae and fewer screws as well as a significant reduction in length of surgery and intraoperative blood loss compared to open surgery in patients with unstable injuries of the subaxial cervical spine. Neuronavigation with robotic intraoperative imaging acquisition enables a safe and highly accurate screw placement also in the cervical spine. Further research with adequately powered data analysis is required to confirm these findings.

## Data Availability

The datasets are available from the corresponding author on reasonable request and if in accordance with current General Data Protection Regulation (GDPR) guidelines.
